# Differential effects of alkaloids on memory in rodents

**DOI:** 10.1038/s41598-021-89245-w

**Published:** 2021-05-10

**Authors:** Patrick M. Callahan, Alvin V. Terry, Manuel C. Peitsch, Julia Hoeng, Kyoko Koshibu

**Affiliations:** 1 Prime Behavior Testing Laboratories, Inc. (PBTLI), Evans, GA 30809 USA; 2 Department of Pharmacology and Toxicology, Medical College of Georgia, Augusta University, Augusta, GA 30912 USA; 3 PMI R&D, Philip Morris Products S.A., Quai Jeanrenaud 5, 2000 Neuchâtel, Switzerland

**Keywords:** Neuroscience, Cognitive neuroscience

## Abstract

Nicotinic acetylcholine receptors (nAChRs) play a critical role in the neuropharmacology of learning and memory. As such, naturally occurring alkaloids that regulate nAChR activity have gained interest for understanding and potentially improving memory function. In this study, we tested the acute effects of three known nicotinic alkaloids, nicotine, cotinine, and anatabine, in suppressing scopolamine-induced memory deficit in rodents by using two classic memory paradigms, Y-maze and novel object recognition (NOR) in mice and rats, respectively. We found that all compounds were able to suppress scopolamine-induced spatial memory deficit in the Y-maze spontaneous alternation paradigm. However, only nicotine was able to suppress the short-term object memory deficit in NOR, despite the higher doses of cotinine and anatabine used to account for their potential differences in nAChR activity. These results indicate that cotinine and anatabine can uniquely regulate short-term spatial memory, while nicotine seems to have more robust and general role in memory regulation in rodents. Thus, nAChR-activating alkaloids may possess distinct procognitive properties in rodents, depending on the memory types examined.

## Introduction

The cholinergic system of the brain is a critical regulator of attention, memory, and higher-order cognitive processing, and its deficits are central to the etiology of dementia^1^. As such, nicotinic acetylcholine receptors (nAChRs) have gained much interest as a target of drug development^2^. Neuronal nAChRs are pentameric proteins composed of various combinations of α (α2–α9) and β (β2–β4) nAChR subunits, differentially expressed throughout the nervous system. Their homomeric (e.g., α7) or heteromeric (e.g., α4β2, α3β4, and α6β2β3) assembly generates multiple nAChR subtypes, which differ in their pharmacological and biophysical properties, such as sensitivity and rate of desensitization^3–6^. High densities of α4β2 and α7 nAChRs, in particular, can be found in brain regions critical for memory, including prefrontal cortex and hippocampus, while α6 containing nAChRs are more commonly found in other structures, such as striatum, substantia nigra, and locus coeruleus^5,7–10^. This diversity of nAChR subtypes allows fine orchestration of neural network activities necessary for proper memory formations^5,11^.

Alkaloids are a class of naturally occurring organic nitrogen-containing bases with various neurological effects in humans and other animals^12–14^. More than 3000 alkaloids have been identified with diverse chemical structures and pharmacological actions^13,15–17^. In particular, pyridine alkaloids that target nAChRs are of great interest due to the critical role they play in neuropharmacology of memory^13,18^. Although nicotine analogs and other pyridine alkaloids have shown a certain degree of toxicity in clinical studies^19–21^, they have also demonstrated potential modulatory effects in various neurological conditions, including memory deficit, particularly in nonclinical animal models^13,22–25^. Among them, nicotine is one of the most well-known natural alkaloid that can be found in many plants of the Solanaceae family with well-established activities on nAChRs^18,26^. A number of studies have reported efficacy of nicotine in regulating memory, such as working memory and recognition memory, in rodents and humans^2,23,24,27–29^. The rodent studies have primarily focused on memory improvements using, for example, radial-arm maze, passive avoidance, and water maze^2,30^. Recent studies have demonstrated that α4, β2, and/or α7 subunit-containing nAChRs participate in the cognitive-enhancing effects of nicotine^9,24^. Although some controversies remain regarding the nootropic effect of nicotine on specific memory functions and on individual differences in such effects, the preponderance of evidence from nonclinical animal and human studies supports memory-enhancing effects as a clinically relevant dimension of nicotine psychopharmacology^9^. In contrast, the effects of other alkaloids from the same chemical class in Solanaceae plants, such as cotinine and anatabine, are less well known^23,31–39^. The main findings for cotinine (the major metabolite of nicotine) include, for example, fear memory extinction, working memory, and sensory gating in rodent models of memory deficit^23,40–45^. The results of these studies suggest that the neurobehavioral effects of cotinine seem to significantly differ from those of nicotine^46–48^. Similarly, a few studies that investigated the effects of anatabine on memory used rodent models of neurodegeneration, such as Alzheimer’s disease and mild traumatic brain injury, the result of which may be at least partly attributed to the anti-inflammatory property of anatabine instead of its nootropic effect^33,49,50^.

In this study, we aimed to investigate these three alkaloids from the same chemical family—nicotine, cotinine, and anatabine—in parallel to understand and compare their potential role in memory. Nicotine served as an ideal, well-established, natural memory enhancing nicotinic reference compound with a very similar chemical structure as cotinine and anatabine. We selected Y-maze spontaneous alteration and novel object recognition (NOR) tasks in rodents to understand their efficacy in regulating spatial and recognition memory. The Y-maze test takes advantage of the innate investigative nature of rodents to explore new environment to assess short-term spatial memory. The test has been shown to be sensitive to hippocampal damage, gene manipulations, and amnesic drugs, for example^51–53^. Similarly, NOR relies on the innate investigative nature of rodents to explore new object to assess recognition memory. Recognition memory is a type of episodic memory that is often reported to be degraded with age in humans and in patients with Alzeheimer’s disease^2^. The rodent spontaneous NOR task has become a particularly popular method for evaluating nicotinics as well as other nootropic drugs^2^. From a practical point of view, both Y-maze and NOR are quite efficient, requiring no or short training, respectively. Neither of them require aversive conditioning, such as footshocks, food deprivation, or water immersion, which reduces the influence of sensory modalities and stress and thus, may simulate the natural memory challenges experienced by humans better than memory paradigms requiring aversive conditioning during the training sessions.

We induced a memory deficit using the muscarinic receptor antagonist scopolamine, which is a standard reference chemical for inducing memory deficit in rodents and humans to mimic the memory decline observed during natural aging and in Alzheimer’s disease patients^54,55^. For example, scopolamine-induced memory deficit in NOR has been used to assess the effect of nicotine and other nicotinic ligands on memory function^56–58^. The efficacy of the nicotinic alkaloids in the indicated memory paradigms was then compared.

## Materials and methods

### Chemicals

**Figure 1:**
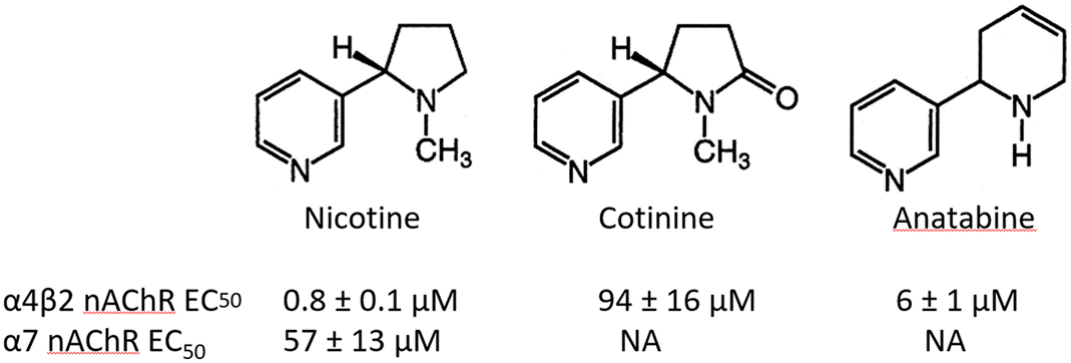
α4β2 and α7 nAChR EC_50_ values of nicotine, cotinine, and anatabine. α4β2 and α7 nAChRs EC_50_ values were determined in Chinese hamster ovarian (CHO) cells overexpressing the respective human nAChRs as reported by Alijevic et al.^18^. Nicotine and anatabine are potent, while cotinine is a weak α4β2 nAChR agonist. Nicotine is also a weak α7 agonist. *NA* not available due to no or low activity.

(‒)-Nicotine free base (CAS No. 54-11-5), (‒)-cotinine free base (CAS No. 486-56-6), and (‒)-scopolamine hydrobromide (CAS No. 6533–68‒2) were purchased from Sigma-Aldrich (St. Louis, MO, USA). (±)-Anatabine free base (purity > 95% by HPLC) was custom-synthesized by WuXi AppTec (Shanghai, China). The nAChR agonist activities of nicotine, cotinine, and anatabine are indicated in Fig. 1 for reference^18^.

### Animals

Adult male Swiss mice (6–7 weeks old; JANVIER, Saint Berthevin, France) were used for the Y-maze spontaneous alternation paradigm test, conducted by Amylgen SAS (Montferrier-sur-Lez, France). The mice were group-housed (4–8 mice per cage) in a temperature (22 ± 2 °C)- and humidity (40–60%)-controlled animal facility with a 12-h light/dark cycle (7:00 a.m. to 7:00 p.m.) and free access to food and water in accordance with the Direction Régionale de l'Alimentation, de l'Agriculture et de la Forêt du Languedoc-Roussillon (agreement #A 34-169-002). During the experiment, animal health was monitored for general appearance, activity, and acute or delayed mortality. All animal procedures were conducted in strict adherence to the European Union Directive of September 22, 2010 (2010/63/UE).

Adult male Wistar rats (3 months old; Envigo, Indianapolis, IN, USA) were used for the NOR paradigm test, conducted by Prime Behavior Testing Laboratories Inc. (PBTLI; Evans, GA, USA). The rats were double-housed in polycarbonate cages with corncob bedding in a vivarium of constant temperature (21–23 °C) and humidity (40–50%) with a 12 h light/dark cycle (7:00 a.m. –7:00 p.m.) and free access to food and water. All animal procedures employed by PBTLI were reviewed and approved by the Institutional Animal Care and Use Committee of Augusta University and conducted in accordance with the Association for Assessment and Accreditation of Laboratory Animal Care International guidelines. Measures were taken to minimize pain or discomfort in accordance with the Guide for the Care and Use of Laboratory Animals^59^. The animal studies were carried out in compliance with the ARRIVE guidelines.

### Y-maze

Adult male Swiss mice (n = 12 per condition) received an intraperitoneal (i.p.) injection of nicotine (0.125, 0.25, and 0.5 mg/kg body weight [b.w.]), cotinine (0.25, 0.5, and 1 mg/kg b.w.), anatabine (0.25, 0.5, and 1 mg/kg b.w.), or vehicle (saline). The highest dose chosen for each of the compounds for Y-maze were the highest tolerated dose assessed in the tolerability test (Supplementary File S1). After 20 min, the mice were subcutaneously (s.c.) injected with scopolamine (0.5 mg/kg b.w. in saline). The injection volume was 5 mL/kg b.w. At 20 min after scopolamine injection, the animals were tested for spontaneous alternation performance in the Y-maze.

The Y-maze test, an index of short-term spatial memory, was designed in accordance with Itoh et al. and Hiramatus & Inoue^60,61^. In brief, the mice were placed at the end of one arm of a Y-shaped maze and allowed to move freely during an 8-min session. The series of arm entries were visually scored by an experimenter blind to the treatment. An alternation was defined as entries into all three arms on consecutive occasions. The number of maximum alternations equaled the total number of arm entries minus two, and the percentage alternation was calculated as (actual alternations/maximum alternations) × 100. The percentage alternation (memory index) and total number of arm entries (exploration index) were then analyzed^62–64^. For graphical representations of the results, memory index was normalized to Veh/Veh controls. None of the animals exhibited extreme behavior (i.e., alternation percentage < 20% or > 90% or number of arm entries < 10). Thus, all animals were included in the analysis. All test compounds were tolerated well at all doses. Experimenters were blind to the test conditions.

### NOR

The NOR task was adapted from Ennaceur & Delacour as previously published by PBTLI^44,65–67^. In brief, adult male Wistar rats were acclimated, weighed, and individually placed in a dimly lit (10 lx) training/testing chamber without any objects for 10 min. Approximately 24 h after the habituation session, the rats (n = 8 per condition) received i.p. injections of scopolamine (0.2 mg/kg b.w.) and nicotine (0.03, 0.1, and 0.3 mg/kg b.w.), cotinine (30 and 100 mg/kg b.w.), anatabine (0.3, 1, and 3 mg/kg b.w.), or vehicle (saline). The doses were selected based on previous publications that showed nootropic effects of the respective compounds in rodents^33,44,65,68,69^. In particular, we chose higher doses of cotinine based on a previously published NOR study where the most significant pro-cognitive effect of cotinine was observed at 10 mg/kg when combined with donepezil and no effect at 0.3 or 1 mg/kg^44^. The injection volume was 1.0–2.0 mL/kg b.w. After 30 min, the rats were placed in the chamber with their nose facing the center of a long wall and allowed to explore two identical objects for 10 min and then returned to their home cages. Object recognition memory was assessed 3 h later by placing the animals back in the chamber with one object identical to that in training (familiar) and one new object (novel). The animals were allowed to freely explore the objects for 5 min. Two plastic multicolored Duplo-Lego block configured towers (12 cm in height, 6 cm in width) or two ceramic conical-shaped green Christmas tree salt/pepper shakers (12 cm in height, 5 cm in diameter) were used as the training objects. The object type that was not used during the training was used during the test session as the novel object as described in Terry et al. and Poddar et al.^44,70^. The primary behavioral measure was the time spent investigating each object, which was defined as the time the animal spent touching the object or directing its nose to the object at a distance of less than or equal to 2 cm or rearing up against the object. However, physically climbing on the object, using the object to support itself, or digging at the base of the object was not considered as an appropriate object exploratory behavior^44,70^.

A discrimination ratio (d2) was then calculated in each test trial and defined as the difference in time spent exploring the novel and familiar objects divided by the total exploration time for both objects: d2 ratio = (novel – familiar)/(novel + familiar). For data inclusion, a rat had to explore each individual object for at least 4 s and spend a minimum of 12 s on total object exploration. All rats met this criteria, and thus, all rats were included in the analysis. All test compounds were tolerated well at all doses. Experimenters were blind to the test conditions.

### Statistical analysis

One-way analysis of variance (ANOVA) with Bonferroni post-hoc multiple comparison test was used for Y-maze statistical analysis (GraphPad Prism v8.2.1, GraphPad Software, San Diego, CA, USA). For the d2 ratio comparisons in NOR, a one-way ANOVA followed by a Student Newman Keuls post-hoc test by using SigmaPlot 11.0 (Systat Software Inc., San Jose, CA, USA) was conducted. The two-way repeated measures ANOVA was applied to the analysis of exploration times for NOR to understand the effects of treatment (or dose), object type (novel vs. familiar), and the treatment by object type interactions. Values with p < 0.05 were considered as statistically significant. Data are expressed as mean ± standard error of the mean (SEM).

## Results

**Figure 2:**
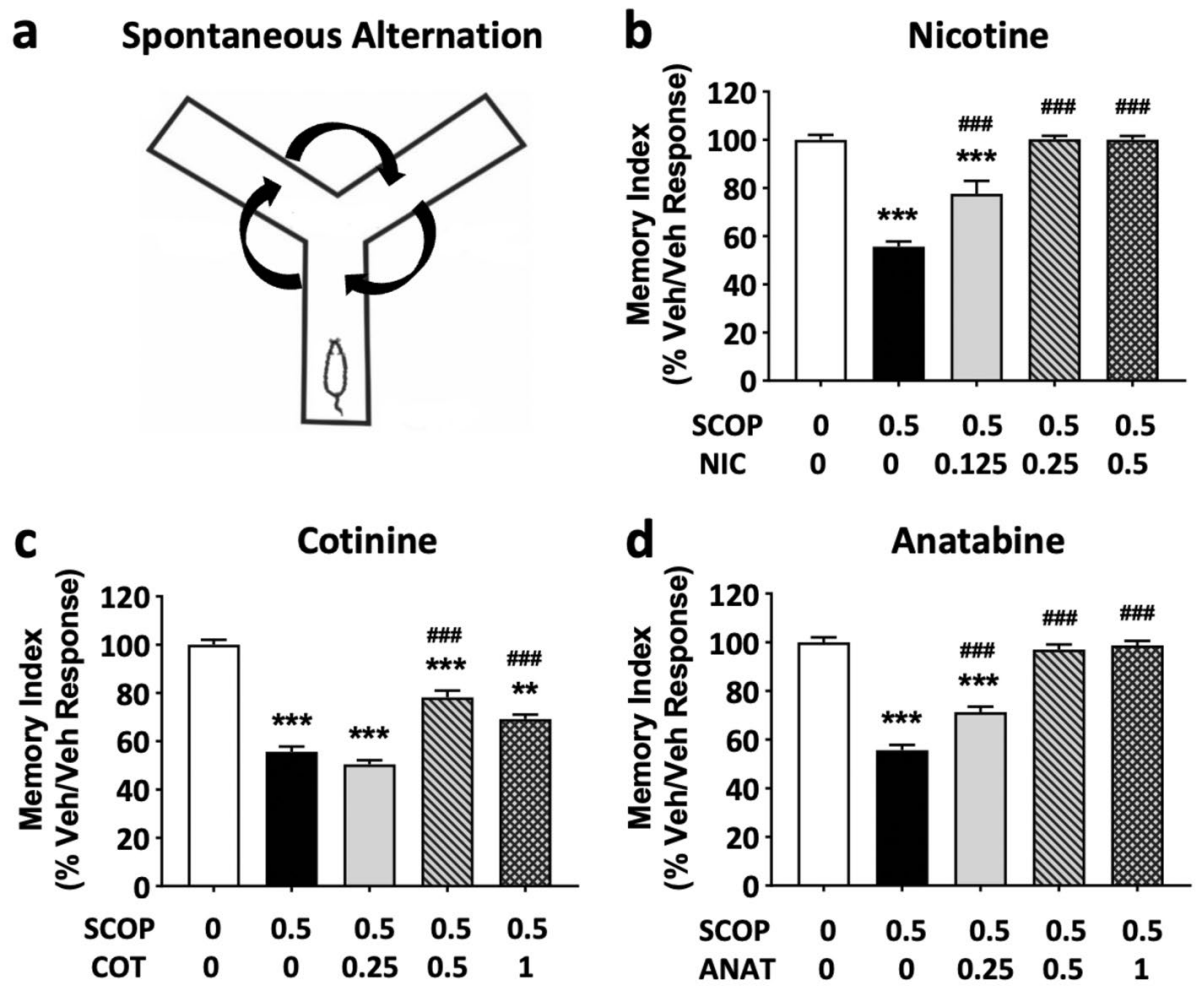
Effects of alkaloids on Y-maze working memory. (**a**) Schematic diagram of the Y-maze spontaneous alternation is shown. The test duration was 8 min. Memory indices, expressed as a percentage of vehicle response (Veh/Veh), are presented for (**b**) nicotine (NIC), (**c**) cotinine (COT), and (**d**) anatabine (ANAT). All compounds were able to significantly suppress scopolamine (SCOP)-induced memory deficit. Doses are indicated below each graph in mg/kg b.w. The vehicle in all panels are the same due to the fact all compounds were tested at the same time. **p < 0.01 and ***p < 0.001 compared with vehicle control without scopolamine (Veh/Veh; white bars). ^###^p < 0.001 compared with vehicle control with scopolamine (black bars). Grey bars are the lowest, bars with slashes are the medium, and checkered bars are the highest concentrations tested of the respective compounds. Data are expressed as mean ± SEM.

In the Y-maze spontaneous alternation test, the ability of mice to remember the previously visited arm was measured as a parameter of functional spatial memory. A single injection of nicotine, cotinine, and anatabine significantly suppressed the scopolamine-induced memory deficit in this spatial memory task in a dose-dependent manner (Fig. 2; treatment effect: p < 0.001). Nicotine and anatabine showed the most potent effect, being able to fully suppress the effect of scopolamine at 0.25 and 0.5 mg/kg b.w. for nicotine and at 0.5 mg/kg and 1 mg/kg b.w. for anatabine (Fig. 2b,d; p < 0.001 compared to scopolamine only treatment for both compounds at both doses). Cotinine also suppressed the effect of scopolamine at 0.5 and 1 mg/kg b.w. (p < 0.001 and p < 0.01, respectively, compared to scopolamine only treatment for both doses) and not at all at 0.25 mg/kg b.w. (Fig. 2c). The lowest dose of nicotine and anatabine and higher two doses of cotinine improved object recognition that was significantly different from Veh/Scopolamine controls, yet still significantly less than a full performance by Veh/Veh controls (bars in Fig. 2 with both asterisks and hash marks). This observation suggests that the animals did not fully recover from scopolamine-induced memory deficit at these particular doses for the respective compounds.

**Figure 3:**
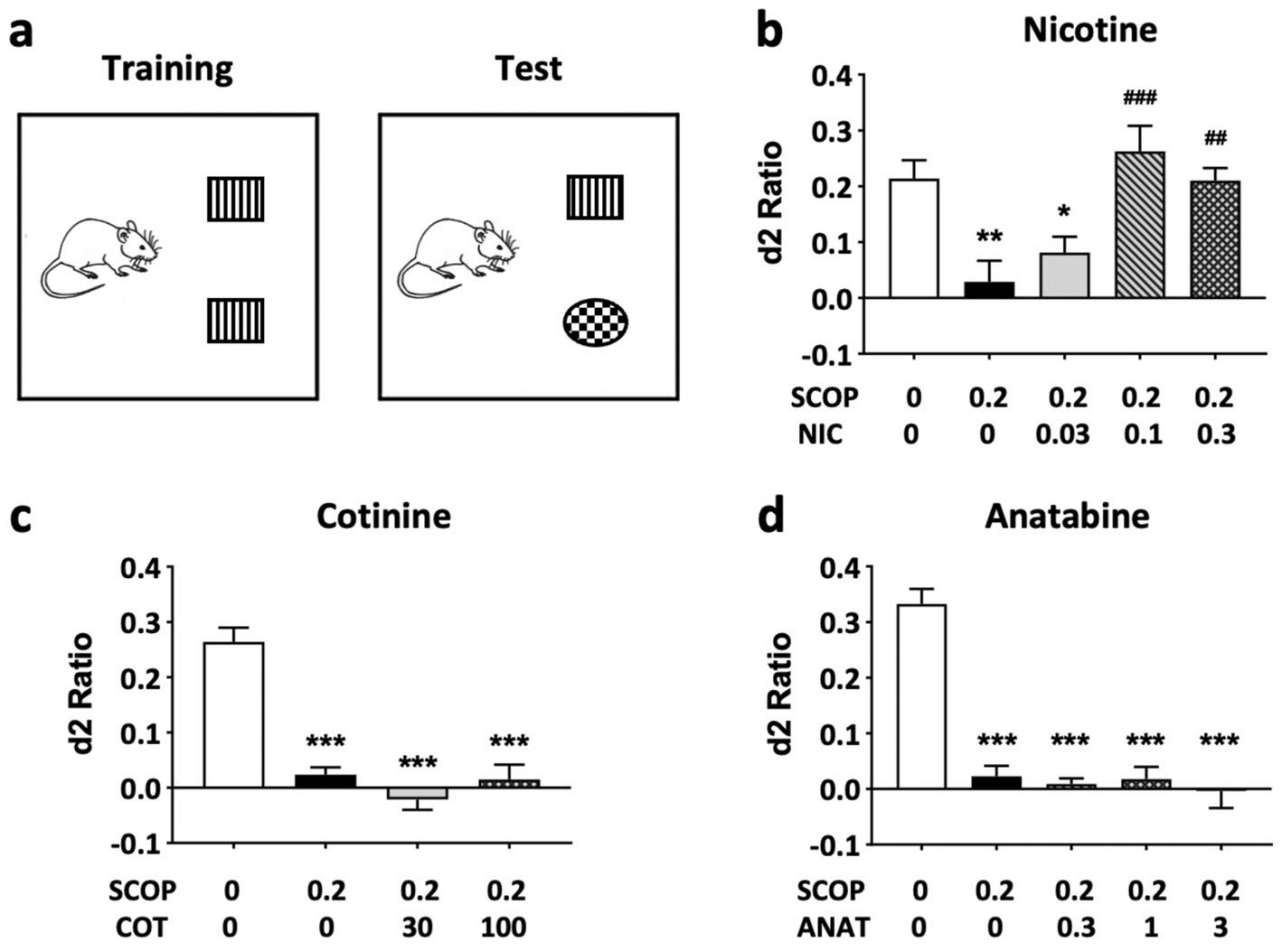
Effects of alkaloids on NOR short-term memory. (**a**) Schematic diagram of the NOR test is shown. The familiar objects are indicated as squares with stripes, and the new object is indicated as a checkered round object for presentation purposes. The test was conducted 3 h after training. The discrimination ratios (d2) are presented for (**b**) nicotine (NIC), (**c**) cotinine (COT), and (**d**) anatabine (ANAT). Only nicotine was able to significantly suppress scopolamine (SCOP)-induced memory deficit at 0.1 and 0.3 mg/kg. Doses are indicated below each graph in mg/kg b.w. *p < 0.05, **p < 0.01, and ***p < 0.001 compared with vehicle control without scopolamine (white bars). ^##^p < 0.001 and ^###^p < 0.001 compared with vehicle control with scopolamine (black bars). Grey bars are the lowest, bars with slashes are the medium, and checkered bars are the highest concentrations tested of the respective compounds. Data are expressed as mean ± SEM.

The effects of these chemicals on short-term object memory were also assessed by the NOR test in rats. In contrast to the results obtained in the Y-maze test of spatial memory, only nicotine was effective in suppressing the memory deficit induced by scopolamine (Fig. 3 and Supplementary File S2; treatment effect: p < 0.001 for the d2 ratio and treatment x object type interaction: p < 0.001 for the exploration time). Nicotine was able to fully suppress the effect of scopolamine at 0.1 and 0.3 mg/kg b.w. (Fig. 3b; p < 0.001 and p < 0.01, respectively). Cotinine and anatabine had no significant effect at tested doses (Fig. 3c,d). All rats explored each individual object for at least 4 s and demonstrated a minimum total object exploration time of 12 s, meeting the inclusion criteria for the study. Both right and left objects were explored equally during the training sessions, and the total exploration times were similar in the training and test sessions regardless of the treatment conditions (data not shown). Therefore, the observed treatment effects in the NOR test sessions were related to differences in recognition memory and not due to the confounding non-cognitive behavioral effects of scopolamine or the test compounds.

## Discussion

In this study, we tested the acute effects of three nAChR-activating alkaloids—nicotine, cotinine, and anatabine—on two types of memory: spatial memory and object recognition memory. We report, for the first time, that cotinine and anatabine differentially regulated spatial and object recognition memory in rodents. Nicotine, on the other hand, suppressed both scopolamine-induced memory deficit in the Y-maze memory task and the NOR short-term memory task in rodents, suggesting a more general role of nicotine in nonclinical models of memory function. This is the first time that these three alkaloids have been tested in parallel to observe their differential effects on specific memory classes in rodents. Previous animal behavioral studies have mainly focused on the effect of nicotine only, which have generally shown memory improvements using, for example, radial-arm maze, passive avoidance, and water maze in rodents^2,30^. The effect of cotinine (the major metabolite of nicotine) on the other hand, is less well-studied and findings are rather specific to, for example, fear memory extinction, working memory, and sensory gating in rodent models of memory deficit^23,40–45^. The results of these studies indirectly suggested that the neurobehavioral effects of cotinine significantly differ from those of nicotine when cotinine is used as a pharmacological tool^46–48^. Similarly, a few studies that investigated the effects of anatabine on memory have shown memory improvement in rodent models of memory deficit, such as Alzheimer’s diseases and mild traumatic brain injury, which may be partially explained by the anti-inflammatory properties of anatabine^33,49,50^. Thus, no direct comparison has been made among these alkaloids in the past.

The reason for the alkaloid-specific effects on memory is unclear, but it is most probably related to the fact that the alkaloids have differential activities on specific subtypes of nAChRs^18^. Previous studies have demonstrated distinct roles of nAChR subtypes in memory processes in rodents^27,71^. For example, α4β2 and α7 nAChRs are the major nAChRs present in the brain, and studies have demonstrated their important regulatory roles in various aspects of memory functions in human and nonclinical studies^2,7^. Although the expression patterns of α4β2 and α7 nAChRs overlap within the key brain regions for memory-associated processes (e.g., hippocampus and prefrontal cortex), there are crucial differences in substructure, cell type, and cellular localization (e.g., pre- vs post-synaptic) specificity between the two nAChR subtypes reported in rodents^5,72,73^. In turn, these finer distributions of the receptors are hypothesized to induce distinct effects on memory. For example, α7 nAChRs in the frontal cortex are thought to be involved in spatial working and reference memory in rats, while α4β2 nAChRs are thought to be involved mainly in spatial working memory^74^. However, in a study that analyzed the ventral hippocampus, the α4β2 nAChR antagonist dihydro-β-erythroidine induced both working and reference memory deficits, while the α7 nAChR antagonist methyllycaconitine only affected the spatial memory in the radial arm maze in rats, indicating a distinct task- and brain region-specific role of these receptors^75^. More recently, a study showed that, in the medial prefrontal cortex, α7 nAChRs are critical for encoding, while α4β2 nAChRs are required for retrieval of object recognition memory in rats^7^. However, neither receptor was involved in spatial memory. The observed effects were attributed to α7 nAChR-mediated glutamatergic pyramidal cell activation, resulting in long-term synaptic potentiation, and α4β2 nAChR-mediated GABAergic interneuron activation, resulting in long-term synaptic depression of hippocampal–prefrontal synapses^7^.

Interestingly, nicotine, cotinine, and anatabine have been reported to differentially bind to and activate α4β2 and α7 nAChRs in vitro^18,26^. Nicotine can, in fact, potently activate α4β2 nAChRs expressed in CHO cells at concentrations 1/10th of anatabine or 1/100th of cotinine concentrations. In addition, nicotine can also weakly activate α7 nAChRs expressed in CHO cells, while cotinine and anatabine cannot^18^. An α7 nAChR-positive allosteric modulator-like activity was previously suggested for cotinine^44^. However, the electrophysiological evidence was rather weak and could not be confirmed by Alijevic et al.^18^. Thus, these subtle differences in the receptor pharmacology of the three alkaloids may contribute to their unique effects on specific classes of memory. In fact, even slight differences in chemical structures are known to have significant changes in receptor pharmacology as exemplified by intense research effort on structure–activity relationships for drug discovery programs^76^ in addition to efforts to improve translatability of in vitro findings to animal studies^77^.

It is worth noting that two different species were used for Y-maze and NOR paradigms. Selections of strains/species of animals used in each test were based on the previous reports, indicating appropriateness and sensitivities of the strains/species to the respective behavioral paradigms^44,78,79^, which were validated by the respective behavioral test facilities. The fact that we were able to detect the effect of all compounds in Y-maze using Swiss mice suggest that the choice of strain and specie was appropriate for testing nicotinic compounds for this particular behavioral paradigm. We chose to use Wistar rats due to the fact that it was the rodent type used in Terry et al. where cotinine was reported to show procognitive effect when combined with donepezil by using NOR^44^. In fact, Wistar rats was reported to perform well in NOR compared to other strains or species^79^ and for testing nicotine^2^. The differences between pharmacokinetics of nicotine between rats and mice have been previously reported with a half-life of plasma nicotine in mice being shorter (less than 10 min in general) than that of rats (approximately 1 to 2 h) after i.p. or subcutaneous administration due to the specie differences in nicotine metabolism^68,80–82^. However, the fact that similar doses of nicotine suppressed scopolamine-induced memory deficit in both NOR and Y-maze in rats and mice, respectively, in the present study suggests that the specie differences in pharmacokinetics or any other variables introduced by the two testing facilities did not play a significant role in this instance. Instead, it supports the robustness of nicotine findings despite of these variables. In fact, Mohler et al. have demonstrated that effects of a compound can be confirmed across sites despite of varying testing equipment, animal suppliers, and general husbandry parameters used across sites^83^, which further supports the validity of our methods and robustness of the findings. Nevertheless, the results of our study should be understood with caution that different species were used in two test facilities.

Together, these findings confirm previous reports that nicotine can regulate cognitive functions in nonclinical animal models^24,84,85^, although it is also not risk free. We hypothesize that spatial and object recognition memory may recruit distinct neural processes that are regulated by specific subtypes of nAChRs in rodents. This specialization of receptor subtypes likely played a part in the memory-type-specific effect of the three alkaloids investigated in the current study in rodent models of memory deficit. The findings presented here is not directly translatable to human findings without additional studies^86^. Although it was out of scope of this paper, investigations on the effects of these and other alkaloids on long-term memory may provide further insights regarding the memory enhancing potential of these natural compounds that may pose less harm than nicotine.

## Supplementary Information

Supplementary File S1.

Supplementary File S2.
