# Supplementary material for: Differential effects of alkaloids on memory in rodents
Source: Sci Rep. 2021 May 10;11:9843. doi: 10.1038/s41598-021-89245-w (PMC8110766; doi:10.1038/s41598-021-89245-w)
Supplement: Supplementary file 1 — Supplementary File S1. [file 41598_2021_89245_MOESM1_ESM.pdf]

Supplementary File S1. Tolerability assessment using functional observational battery in mice

Mean tolerability scores for nicotine (0.3, 1, and 3 mg/kg b.w.), cotinine (3, 10, and 30 mg/kg b.w.), and anatabine (1, 3, and 10 mg/kg b.w.) are presented. The tolerability observations were made immediately after the injection (H1), then at 1 h (H2), 2h (H3), 3h (H4), 4h (H5), and 5h (H6). Score of 0 to 4 (0 = none; 1 = slight; 2 = moderate; 3 = marked; 4 = extreme) was assigned to each mouse per compound dose per parameter per time point. N = 3 mice.

Grading score		0 = none		1 = slight		2 = moderate		3= marqued		4= extreme
H1		Nicotine 0.3 mg/kg	Nicotine 1 mg/kg	Nicotine 3 mg/kg	Cotinine 3 mg/kg	Cotinine 10 mg/kg	Cotinine 30 mg/kg	Anatabine 1 mg/kg	Anatabine 3 mg/kg	Anatabine 10 mg/kg
Body position	Flattened									
	Lying on side	0	3.3	4	1	1	0.3	0	1.6	3
	Sitting									
	Standing									
Locomotor activity	Scratch									
	Spatial duration/speed	0	0	0	0.3	1	0.3	0	0	0
Abnormal activity		0	0	0	0.3	0	0	0	0	0
Convulsions		0	0	0	0	0.3	0	0	0	1
Piloerection		0	0.3	1.33	0.6	0.3	0	0.6	1	0.3
Diarrhea/urination		0	0.6	0.6	0.3	0	0	0	0	0
Salivation		0	0	0	0	0	0	0	0	0
Tremors		0	0	0	0	0	0	0	0	0
Palpebral closure		0	0	0	0	0	0	0	0	0
Reactivity/sensory function		0	0	0	0	0	0	0	0	0
Grip strength		0	0	0	0	0	0	0	0	0
Abnormal breathing		0	2.3	3.33	1.6	1.33	1.33	0.3	2	3
Vocalization		0	0	0.33	0.3	0	0	0	0	0
Lacrimation		0	0	0	0	0	0	0	0	0

H2		Nicotine 0.3 mg/kg	Nicotine 1 mg/kg	Nicotine 3 mg/kg	Cotinine 3 mg/kg	Cotinine 10 mg/kg	Cotinine 30 mg/kg	Anatabine 1 mg/kg	Anatabine 3 mg/kg	Anatabine 10 mg/kg
Body position	Flattened									
	Lying on side	0	0	0.3	0.3	0	0	0	0	0.3
	Sitting									
	Standing									
Locomotor activity	Scratch									
	Spatial duration/speed	0	0	0	0	0	0	0	0	0
Abnormal activity		0	0	0	0	0	0	0	0	0
Convulsions		0	0	0	0	0	0	0	0	0
Piloerection		0	0	1	0.3	0	0	0	0	0.3
Diarrhea/urination		0	0	0	0	0	0	0	0	0
Salivation		0	0	0	0	0	0	0	0	0
Tremors		0	0	0	0	0	0	0	0	0
Palpebral closure		0	0	0	0	0	0	0	0	0
Reactivity/sensory function		0	0	0	0	0	0	0	0	0
Grip strength		0	0	0	0	0	0	0	0	0
Abnormal breathing		0	0	0.6	0.6	0	0	0	0	0.3
Vocalization		0	0	0	0	0	0	0	0	0
Lacrimation		0	0	0	0	0	0	0	0	0

H3		Nicotine	Nicotine	Nicotine	Cotinine	Cotinine	Cotinine	Anatabine	Anatabine	Anatabine
		0.3 mg/kg	1 mg/kg	3 mg/kg	3 mg/kg	10 mg/kg	30 mg/kg	1 mg/kg	3 mg/kg	10 mg/kg
Body position	Flattened									
	Lying on side	0	0	0	0.3	0	0	0	0	0
	Sitting									
	Standing									
Locomotor activity	Scratch									
	Spatial duration/speed	0	0	0	0	0	0	0	0	0
Abnormal activity		0	0	0	0	0	0	0	0	0
Convulsions		0	0	0	0	0	0	0	0	0
Piloerection		0	0	0	0.3	0	0	0	0	0
Diarrhea/urination		0	0	0	0	0	0	0	0	0
Salivation		0	0	0	0	0	0	0	0	0
Tremors		0	0	0	0	0	0	0	0	0
Palpebral closure		0	0	0	0	0	0	0	0	0
Reactivity/sensory function		0	0	0	0	0	0	0	0	0
Grip strength		0	0	0	0	0	0	0	0	0
Abnormal breathing		0	0	0	0.3	0	0	0	0	0
Vocalization		0	0	0	0	0	0	0	0	0
Lacrimation		0	0	0	0	0	0	0	0	0

H4		Nicotine	Nicotine	Nicotine	Cotinine	Cotinine	Cotinine	Anatabine	Anatabine	Anatabine
		0.3 mg/kg	1 mg/kg	3 mg/kg	3 mg/kg	10 mg/kg	30 mg/kg	1 mg/kg	3 mg/kg	10 mg/kg
Body position	Flattened									
	Lying on side	0	0	0	0	0	0	0	0	0
	Sitting									
	Standing									
Locomotor activity	Scratch									
	Spatial duration/speed	0	0	0	0	0	0	0	0	0
Abnormal activity		0	0	0	0	0	0	0	0	0
Convulsions		0	0	0	0	0	0	0	0	0
Piloerection		0	0	0	0.3	0	0	0	0	0
Diarrhea/urination		0	0	0	0	0	0	0	0	0
Salivation		0	0	0	0	0	0	0	0	0
Tremors		0	0	0	0	0	0	0	0	0
Palpebral closure		0	0	0	0	0	0	0	0	0
Reactivity/sensory function		0	0	0	0	0	0	0	0	0
Grip strength		0	0	0	0	0	0	0	0	0
Abnormal breathing		0	0	0	0.3	0	0	0	0	0
Vocalization		0	0	0	0	0	0	0	0	0
Lacrimation		0	0	0	0	0	0	0	0	0

H5		Nicotine	Nicotine	Nicotine	Cotinine	Cotinine	Cotinine	Anatabine	Anatabine	Anatabine
		0.3 mg/kg	1 mg/kg	3 mg/kg	3 mg/kg	10 mg/kg	30 mg/kg	1 mg/kg	3 mg/kg	10 mg/kg
Body position	Flattened									
	Lying on side	0	0	0	0	0	0	0	0	0
	Sitting									
	Standing									
Locomotor activity	Scratch									
	Spatial duration/speed	0	0	0	0	0	0	0	0	0
Abnormal activity		0	0	0	0	0	0	0	0	0
Convulsions		0	0	0	0	0	0	0	0	0
Piloerection		0	0	0	0.3	0	0	0	0	0
Diarrhea/urination		0	0	0	0	0	0	0	0	0
Salivation		0	0	0	0	0	0	0	0	0
Tremors		0	0	0	0	0	0	0	0	0
Palpebral closure		0	0	0	0	0	0	0	0	0
Reactivity/sensory function		0	0	0	0	0	0	0	0	0
Grip strength		0	0	0	0	0	0	0	0	0
Abnormal breathing		0	0	0	0.3	0	0	0	0	0
Vocalization		0	0	0	0	0	0	0	0	0
Lacrimation		0	0	0	0	0	0	0	0	0

H6		Nicotine 0.3 mg/kg	Nicotine 1 mg/kg	Nicotine 3 mg/kg	Cotinine 3 mg/kg	Cotinine 10 mg/kg	Cotinine 30 mg/kg	Anatabine 1 mg/kg	Anatabine 3 mg/kg	Anatabine 10 mg/kg
Body position	Flattened									
	Lying on side	0	0	0	0	0	0	0	0	0
	Sitting									
	Standing									
Locomotor activity	Scratch									
	Spatial duration/speed	0	0	0	0	0	0	0	0	0
Abnormal activity		0	0	0	0	0	0	0	0	0
Convulsions		0	0	0	0	0	0	0	0	0
Piloerection		0	0	0	0.3	0	0	0	0	0
Diarrhea/urination		0	0	0	0	0	0	0	0	0
Salivation		0	0	0	0	0	0	0	0	0
Tremors		0	0	0	0	0	0	0	0	0
Palpebral closure		0	0	0	0	0	0	0	0	0
Reactivity/sensory function		0	0	0	0	0	0	0	0	0
Grip strength		0	0	0	0	0	0	0	0	0
Abnormal breathing		0	0	0	0.3	0	0	0	0	0
Vocalization		0	0	0	0	0	0	0	0	0
Lacrimation		0	0	0	0	0	0	0	0	0
