# Supplementary figures and images for: Differential effects of alkaloids on memory in rodents
Source: Sci Rep. 2021 May 10;11:9843. doi: 10.1038/s41598-021-89245-w (PMC8110766; doi:10.1038/s41598-021-89245-w)

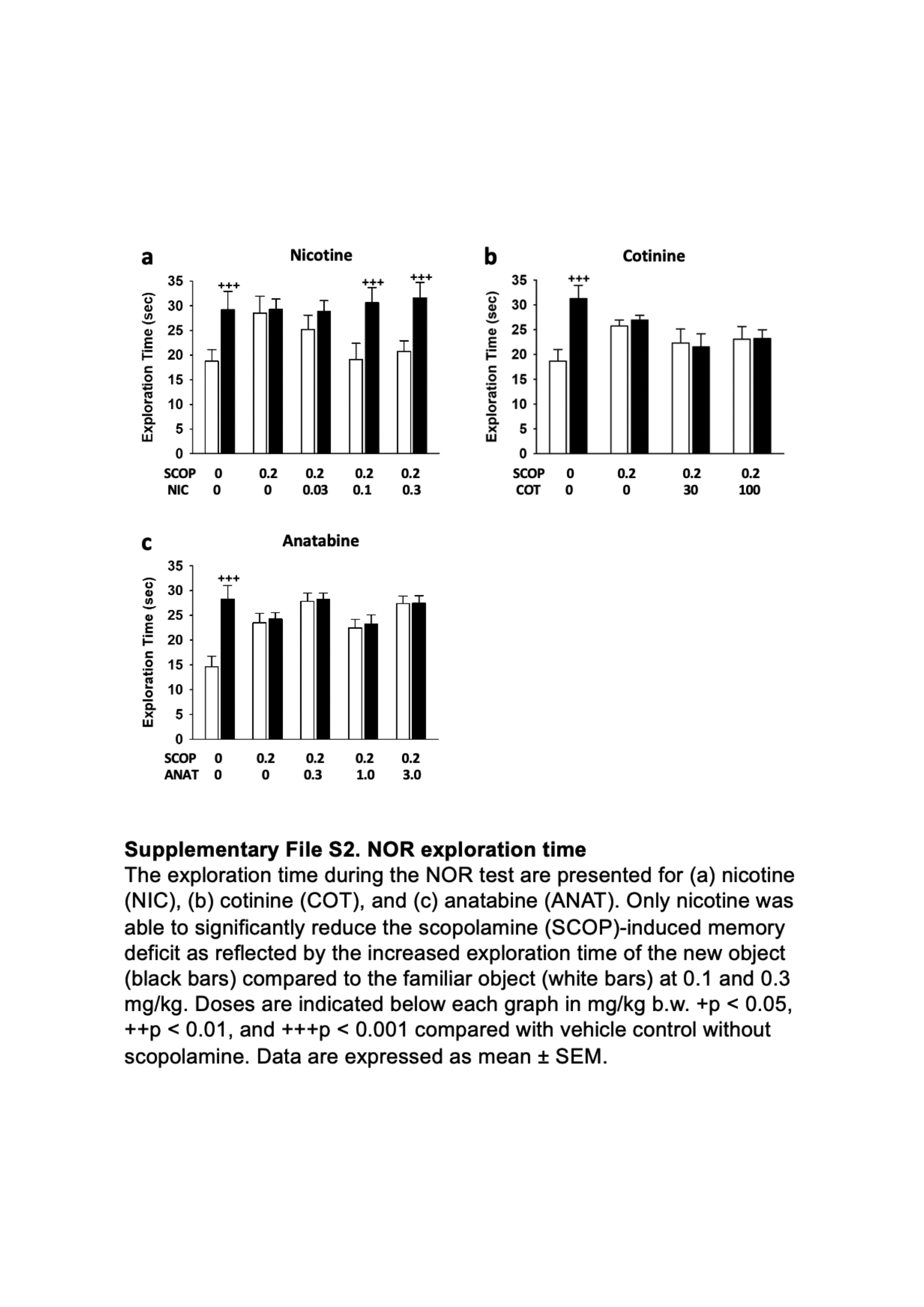

Supplement: Supplementary file 2 — Supplementary File S2. [file 41598_2021_89245_MOESM2_ESM.jpg]
